# Enhanced Healing of Diabetic Wounds by Topical Administration of Adipose Tissue-Derived Stromal Cells Overexpressing Stromal-Derived Factor-1: Biodistribution and Engraftment Analysis by Bioluminescent Imaging

**DOI:** 10.4061/2011/304562

**Published:** 2010-12-26

**Authors:** Giuliana Di Rocco, Antonietta Gentile, Annalisa Antonini, Francesca Ceradini, Joseph C. Wu, Maurizio C. Capogrossi, Gabriele Toietta

**Affiliations:** ^1^Laboratorio di Biologia Vascolare e Medicina Rigenerativa, Centro Cardiologico Fondazione Monzino - IRCCS, Via Parea 4, 20138 Milan, Italy; ^2^Laboratorio di Patologia Vascolare, Istituto Dermopatico dell'Immacolata - IRCCS, Via dei Monti di Creta 104, 00167 Rome, Italy; ^3^Department of Medicine and Radiology, Division of Cardiology, Stanford University School of Medicine, 300 Pasteur Drive, Grant S140B, Stanford, CA 94305-5111, USA

## Abstract

Chronic ulcers represent a major health problem in diabetic patients resulting in pain and discomfort. Conventional therapy does not guarantee adequate wound repair. In diabetes, impaired healing is partly due to poor endothelial progenitor cells mobilisation and homing, with altered levels of the chemokine stromal-derived factor-1 (SDF-1) at the wound site. Adipose tissue-associated stromal cells (AT-SCs) can provide an accessible source of progenitor cells secreting proangiogenic factors and differentiating into endothelial-like cells. We demonstrated that topical administration of AT-SCs genetically modified *ex vivo* to overexpress SDF-1, promotes wound healing into diabetic mice. In particular, by *in vivo* bioluminescent imaging analysis, we monitored biodistribution and survival after transplantation of luciferase-expressing cells. In conclusion, this study indicates the therapeutic potential of AT-SCs administration in wound healing, through cell differentiation, enhanced cellular recruitment at the wound site, and paracrine effects associated with local growth-factors production.

## 1. Introduction

Skin ulcers due to micro/macrovascular disease and peripheral neuropathy represent a common complication in diabetes. Patients suffering from chronic wounds have a diminished quality of life, require frequent hospitalization, and experience increased morbidity and mortality, causing great societal and economical costs [1]. Current treatments, which include relief of pressure at the wound site, aggressive surgical debridement, control of infection, and arterial reconstruction, are limited in effectiveness and often not sufficient to guarantee adequate healing [2]. In fact, a significant number of patients do not respond to conventional therapies, and recurrence of symptoms is frequent. Short protein half-life and inefficient delivery to target cells hamper some nonconventional treatments, including topical application of recombinant growth factors to promote tissue regeneration [3].

Given the increased incidence of the disease, it is necessary to develop improved therapies to treat diabetic ulcers to reduce patient discomfort and lower societal burden. Recently, stem cell application has been suggested as a possible novel therapeutic option to promote chronic wound healing [4]. In particular, mesenchymal stem cells isolated from fetal liver [5] and bone marrow [6, 7] can enhance wound repair through differentiation and angiogenesis promotion. Mesenchymal cells sharing similar characteristics of the ones isolated from bone marrow also can be derived from adipose tissue [8]. Adipose tissue-derived stromal cells (AT-SCs), also known as stromal vascular fraction cells (AT-SVFs), are collected from adipose tissue by collagenase digestion and differential centrifugation [9]. Since liposuction involves only local anaesthesia, cells may be obtained from the patient with a repeatable, nondebilitating operation and autologously transplanted at the site of tissue regeneration. The procedure can be performed in a large-scale, reproducible manner according to GMP regulations, allowing AT-SCs to be used in preclinical studies and experimental clinical trials [10].

Adipose tissue-derived multipotential precursor cells are able to differentiate in several cells types of both mesodermal and nonmesodermal origins, including adipocytes, chondrocytes, osteocytes, myocytes, hepatocytes, endocrine pancreatic cells, neurons, and endothelial cells (for review see [9]).

AT-SCs secrete angiogenic molecules, including HGF, VEGF, PlGF, IGF, and KGF [11, 12]. Moreover, transcriptional profiling has demonstrated the expression of proangiogenic genes [13]. Transplantation of AT-SCs has been shown to promote therapeutic angiogenesis in hind limb ischemia in mice [14–16]. This may be due to integration of AT-SCs into vessels *in vivo* [17] and to the contribution to neovascularisation mediated by a paracrine effect [18]. Moreover, AT-SCs can promote human dermal fibroblast proliferation [12] and wound healing in a mouse model [19, 20] both via direct cell-to-cell contact and by a paracrine effect. In addition, a protective effect of AT-SCs and their secretory factors during oxidative injury has been described [21]. Improved wound healing of adipose tissue extracts was determined in a porcine experimental model [6], while AT-SCs extract did not affect murine wound repair [19].

Taken together, these studies support the development of approaches using AT-SCs administration to promote wound healing. Nonetheless, we know very little about how transplanted cells behave *in vivo*, as well as their ability of engraftment, persistence, differentiation, and biodistribution after *in vivo *administration. *In vivo* studies in animal models have the potential to demonstrate the clinical potential of stem cell therapy by elucidating cell biology and physiology of the transplanted cells and their progeny. Current methods of studying stem cell fate after administration mainly rely on histochemical evaluation of samples obtained from sacrificed animals. This approach is time consuming, laborious, and expensive, requiring sectioning analysis of multiple samples deriving from a large number of experimental animals. To better understand stem cell activity *in vivo*, rapid, affordable, and noninvasive imaging techniques are needed.

In order to evaluate engraftment of AT-SCs in a murine model of diabetic impaired wound healing, we used bioluminescent imaging to track transplanted cells. Furthermore, we were interested in determining whether concomitant overexpression of SDF-1 may further promote wound healing and cell engraftment. In fact, in the diabetic condition, impaired healing is at least in part due to SDF-1 downregulation [22], which affects migration and homing at the wound site of circulating endothelial progenitor cells (EPCs) [23]. On the other hand, exogenous administration at the lesion site of recombinant SDF-1 [24] or of a lentiviral vector expressing SDF-1 [25] reversed the impaired EPC homing in a murine model of diabetic wound healing. Conversely, SDF-1 inhibition in diabetic animals resulted in further impairment of wound repair [26]. Moreover, SDF-1 overexpression increased survival and growth of CXCR4-expressing mesenchymal stem cells both *in vitro* [27] and *in vivo * [28, 29].

Here, we show that topical administration of AT-SCs promotes wound healing in diabetic mice and determine by BLI the biodistribution and the kinetic of engraftment of administered cells.

## 2. Material and Methods

### 2.1. Experimental Animals

Mice used in the study were 6- to 8-week-old wild-type Swiss CD1 males, transgenic mice expressing ubiquitously GFP [30], or GFP and firefly luciferase [31] from colonies maintained in our institutional animal facility. All experimental procedures were performed according to the guidelines of the Italian National Institutes of Health and were approved by the Institutional Animal Care and Use Committee.

Induction of diabetes was obtained by intraperitoneal injection of 50 mg/kg streptozotocin (Sigma-Aldrich, St. Louis, MO) in 0.05 M Na citrate, pH 4.5, for 5 consecutive days. Two weeks after the last streptozotocin injection, animals were fasted for 2 hours and blood glucose level measured using an Ascensia Confirm glucometer (Bayern HealthCare, Basel, Switzerland). Mice with glycaemia above 200 mg/dl were selected for further studies. Three weeks later, diabetic animals were used for wound-healing experiments.

### 2.2. Cells Isolation

AT-SCs were obtained from 6-week-old mice as previously described [32]. Briefly, inguinal subcutaneous fat pads were digested for 45 minutes in a shaking water bath at 37°C in PBS containing 2% BSA and 2 mg/ml collagenase A (Roche Diagnostics, Mannheim, Germany). After tissue disaggregation, cells were filtered through a 40 *μ*m cell strainer (BD Falcon, Franklin Lakes, NJ) and collected by centrifugation at low speed (500 g) to remove floating mature adipocytes. Cells were then washed in PBS, counted, and used for wound healing experiments. With this procedure, we isolated approximately 0.8–1.2 × 10^6^ cells from each 6-week-old mouse.

To obtain adipose-derived adherent stromal (ADAS) [13], sometimes referred to also as adipose issue-derived mesenchymal cells (AT-MSCs) [33], cells were plated in DMEM 20% FCS in tissue culture dishes. After an overnight incubation, the adherent cell population was obtained.

Skin fibroblasts were obtained from the same animals as previously described [34]. Briefly, skin samples were washed in PBS, then the subcutaneous tissues were eliminated and the epidermis removed by enzymatic digestion. After a wash in PBS, dermal samples were cut into small pieces and treated with 0.3% trypsin in PBS for 30 min in a 37°C water bath. Ice-cold complete medium (DMEM 10% FCS) was then added and the samples vigorously mixed on a vortex. The suspension was passed through a 40 *μ*m cell strainer, and cells were collected by centrifugation (10 min at 150 × g, 4°C). Cells were then washed in PBS and counted and either used as control for angiogenesis plug assay (see below) or plated on tissue culture dishes (0.4 × 10^5^ cells/cm^2^) in complete medium.

### 2.3. Construction of Viral Vectors

Recombinant first-generation E1-E3-deleted adenoviral (Ad) vectors for expression of SDF-1 and GFP were obtained using the Ad-Easy system as previously described [35]. After amplification, the adenoviral vectors were purified by CsCl_2_ gradient ultracentrifugation [36] and dialyzed against a solution containing 3% sucrose, 10 mM Tris, pH 7.8, 150 mM NaCl, and 10 mM MgCl_2_. Viral titers were estimated by serial dilution of the viral stocks in 293 cells and expressed as plaque-forming units per ml (PFU/ml). Viral stocks were tested for absence of replication-competent adenovirus on A549 cells.

### 2.4. In Vitro Lesion Repair Assay

The assay was performed as described by Bilic et al. [37] in triplicate. Second passage mesenchymal cells derived from adipose tissue were grown to confluence in complete medium (DMEM 20% FCS). Cells were then transduced by adenoviral-mediated gene transfer for 1 hour at 37°C at multiplicity of infection of 10. Cells were washed with PBS then grown with serum-free medium supplemented with 0.1% bovine serum albumin (BSA). The day after gene transfer cell monolayer was lesioned using a 2 mm cell scraper without damaging the dish surface. Lesion areas were recorded with a digital camera at time zero and after 1 and 2 days. Analysis was performed by tracing the lesion edges and calculating the pixel area using the Image Analysis System (IAS, Delta Sistemi, Rome, Italy). *In vitro* lesion repair is expressed as percent of the value calculated at time zero.

### 2.5. In Vivo Angiogenesis Gel Plug Assay

The assay is based on the implant of Matrigel plugs [38]. Freshly isolated AT-SCs resuspended in PBS were mixed with Cultrex growth factor-reduced basement membrane extract (Trevigen Inc., Gaithersburgh, MD) maintained at 4°C (liquid state). An aliquot of 400 *μ*l of Cultrex containing 7 × 10^5^ cells was injected subcutaneously into Swiss CD1 mice gathered in experimental groups of 5 animals each near the abdominal midline. The solution and the syringe were kept on ice before the injection to prevent gelling in the needle. A week later, mice were sacrificed and gel plugs removed. Samples were fixed in 4% formaldehyde and embedded in paraffin for sectioning and histological analysis. Sections were then processed with Masson Trichromic stain and capillary density determined.

### 2.6. Cell Administration at the Wound Site

Hyperglycaemic animals were sedated by intraperitoneal administration of Avertin (200 mg/kg, Sigma-Aldrich) and shaved on the dorsum, and a full-thickness wound was performed on the dorsal midline using a 3.5 mm-diameter biopsy punch (Stiefel, Offenbach am Main, Germany). Right after wound induction, freshly isolated AT-SCs were then administered site in a single dose (7.5 × 10^5^ cells/mouse dissolved in 40 *μ*l of saline solution) topically at the lesion. Control animals received the same volume of saline solution. Wounded animals were then housed individually.

### 2.7. Wound Analysis

At different time points (0, 3, 5, 7, 10, and 14 days) after wounding, lesion closure was documented using a digital camera. Images were processed and analyzed by tracing the wound margin and calculating the pixel area using the Image Analysis System (IAS). Re-epithelialisation is reported as the percentage of the initial wound area and calculated as re-epithelialisation percentage = [1 − (area on day of analysis/area on day 0)] × 100 [39]. The day in which the full-thickness wound was seen to be completely closed was taken as the day of complete healing. Moreover, images of haematoxylin and eosin stained slides of each wound obtained from maximal cross-sections were digitally acquired, then the epithelial gap (distance between the two epithelial edges) was measured to assess re-epithelialization.

### 2.8. Ex Vivo and In Vivo Optical Bioluminescent Imaging

*Ex vivo *and* in vivo* imaging analysis was performed using the IVIS Lumina from Caliper Life Sciences (Caliper Life Sciences, Hopkinton, MA). For *ex vivo* imaging, AT-SCs isolated from inguinal fad pads from luciferase-expressing mice were plated into clear bottom tissue culture dishes and incubated in a solution of D-luciferin (Caliper Life Sciences) dissolved in prewarmed tissue culture medium (150 *μ*g/ml) before analysis. For *in vivo* analysis, mice were anesthetized with Avertin and D-luciferin dissolved in PBS (150 mg/kg body weight), was administered i.p. 10 minutes before analysis. Photons emitted from luciferase-expressing AT-SCs transplanted into the animals were collected with final accumulation times of 1 to 5 minutes, depending on the intensity of the bioluminescence emission. The same mice were analyzed at different time points after transplantation with the same procedure, providing longitudinal data of transplanted cells biodistribution and survival.

### 2.9. Histological Analysis

Biopsies were fixed in 4% formaldehyde for 48 hours, embedded in paraffin, and serially sectioned (7 *μ*m) perpendicularly to the wound surface. Every eighth section of each wound was haematoxylin and eosin stained to perform morphologic analyses. Immunohistochemistry on formalin fixed, paraffin-embedded tissue was performed with antibodies against GFP (Abcam Ltd., Cambridge, UK).

### 2.10. Statistical Analysis

Results are expressed as means ± SEM. Data analysis and comparisons between control and treated groups were done with INSTAT (GraphPad, San Diego). The significance of differences was assessed with a two-tailed Student *t*-test for unpaired data; statistical significance level was set at *P* < .05.

## 3. Results and Discussion

### 3.1. SDF-1 Overexpression Promotes Lesion Repair in AT-SCs Monolayer Cultures In Vitro

**Figure 1:**
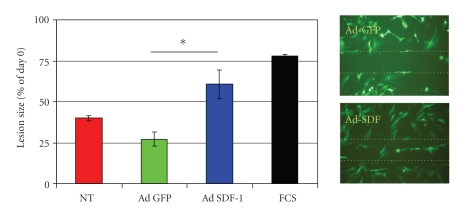
*In vitro lesion repair assay. *Lesion closure (expressed as % of the area at day 0) in an *in vitro* lesion repair assay. Nontransduced AT-SCs (NT) or transduced with an adenoviral vector expressing GFP (Ad-GFP) or expressing SDF-1 and GFP (Ad-SDF-1) were maintained in serum-free medium supplemented by 0.1% BSA. Control cells were grown in complete medium (DMEM-20% FCS). Data are expressed as mean ± SEM, and experiment was performed in triplicate. Statistical significance level was set at *P* < .05. No statistically significant difference was assessed between the NT and Ad-GFP-transduced groups. At 48 hours, lesion repair in the Ad-SDF-1 group was improved in comparison with both NT group (*P* = .018) and Ad-GFP (*P = .006). Representative fluorescence micrographs of lesion area (dotted lines) at day 2. Original magnification 40X.

We determine that AT-SCs have a repair potential in an *in vitro* lesion repair assay performed as previously described [37]. AT-SCs cultured in 20% FCS containing filled a 2 mm gap performed on the cell monolayer in less than 3 days. Non-transduced and mock-transduced AT-SCs maintained in serum-free medium were not able to repair the scratch. Non-transduced cells performed better than mock-transduced (Ad-GFP), possibly for toxicity associated with viral exposure, but the difference was not statistically significant at 48 hours after lesion induction. On the other hand, cells after adenoviral-mediated gene transfer of SDF-1 had an improved lesion repair potential after 48 hours from the lesion, compared both with non-transduced and mock-transduced AT-SCs (Figure 1). 

AT-SCs have been shown to express the CXCR4 alpha-chemokine receptor specific for stromal-derived factor-1, and its overexpression increases AT-SCs migration and proliferation [40]. Accordingly, these results indicate that SDF-1 may act as an autocrine factor on AT-SCs after a viral-mediated gene transfer that promotes cell proliferation and migration.

### 3.2. AT-SCs Promotes In Vivo Angiogenesis in a Gel Plug Assay

**Figure 2:**
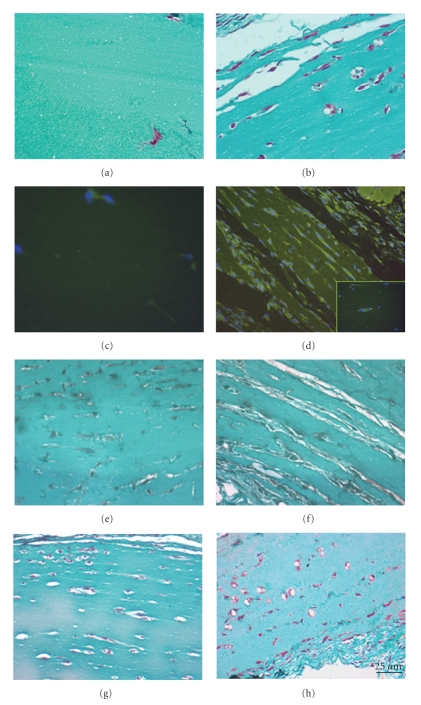
*In vivo angiogenesis gel plug assay. *AT-SCs isolated from ubiquitously GFP-expressing mice were mixed with Cultrex and injected subcutaneously into CD1 recipient. After 1 week, gel plugs were analyzed and capillary density was determined by Masson Trichromic stain (b) and anti-GFP immunohistochemistry (d). Control animals received gel plugs containing saline solution (a)–(c). Masson trichromic stain of implanted gel plugs containing nonconditioned serum-free medium (e) and conditioned medium from AT-SCs cultures (f); AT-SCs were genetically modified by Ad-GFP (g) and Ad-SDF-1 (h). The number of endothelial-like structures formation was slightly improved by SDF-1 overexpression, but the difference versus the control group (Ad-GFP) did not achieve statistical significance (**P**-value ≥.05).

We found that the stromal fraction isolated from adipose tissue contained a population of cells capable of differentiating into endothelial-like structures in a Matrigel plug assay [14]. To perform this assay, we isolated AT-SCs from transgenic mice ubiquitously expressing GFP [30] and then transplanted the plugs containing the GFP-positive cells in GFP-negative animals. We were then able to detect endothelial-like structures derived from implanted GFP-positive AT-SCs (Figure 2(b)), while we failed to detect similar structure in control mice (Figure 2(a)). In this experimental condition, more than 99% of endothelial-like structures were derived from GFP-positive cells (Figure 2(d)). We also performed the assay using AT-SCs transduced with an adenoviral mock vector and AT-SCs transduced with an adenoviral vector expressing SDF-1. We observed that even in normoglycemic conditions, the number of endothelial-like structures formation was slightly improved (5%) by SDF-1 overexpression (Figure 2(h)); albeit in the experimental setting used to perform the analysis (*n* = 5 per group), the difference versus the control group did not achieve statistical significance (*P*-value ≥.05).

Matrigel plugs containing an equal number of skin fibroblasts obtained from the same animals used for isolation of AT-SCs were used as controls and failed to organize into endothelial-like structures (data not shown).

In addition, we also performed a Matrigel plug assay using a conditioned medium obtained from an overnight culture of AT-SCs and found that secreted soluble factors produced by AT-SCs may promote angiogenesis (Figure 2(f)). This is in accordance with several studies which have proved that AT-SCs secrete a panel of angiogenic molecules [21], which have the potential to be, at least in part, responsible for the therapeutic effect we and others have observed on wound healing.

Collectively these sets of data indicate that AT-SCs are able to promote vessel formation *in vivo* in a Matrigel assay by taking part in vessel-like structure organization and by secretion of proangiogenic factors.

### 3.3. AT-SCs Transplantation Promotes Wound Healing in Diabetic Animals

**Figure 3:**
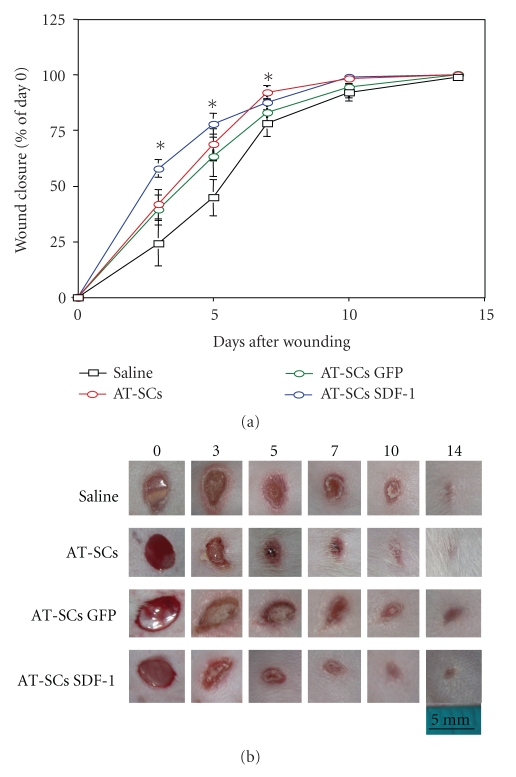
*Wound closure in diabetic animals. *Effect of topical administration of AT-SCs at the lesion site. Four groups of wounded diabetic mice were treated with saline solution (Saline), AT-SCs not genetically modified (AT-SCs) or AT-SCs after adenoviral-mediated gene transfer of GFP (AT-SCs GFP), and SDF-1 (AT-SCs SDF-1). At different time points after wounding, the area of the lesion was determined using image analysis. Re-epithelialization is reported as the percentage of the initial wound area (a). Representative images from an animal from each group were taken immediately after wounding (day 0) and at different time points after injury (b). Data are expressed as mean ± SEM indicated by error bars; *n *= 8–12 wounds for each group. Values of the AT-SCs, AT-SCs GFP, and AT-SCs SDF-1 were significantly different (*P* < .05) when compared to the saline group at the time points indicated by an asterisk. Moreover, at 3 and 5 days, wound closure was improved in AT-SCs SDF-1-treated animals in comparison with the AT-SCs GFP group (*P* < .05).

For *in vivo *studies, we used streptozotocin-induced CD1 diabetic mice receiving a full-thickness wound on the dorsal midline. We observed that topical administration at the lesion site of 7.5 × 10^5^ AT-SCs significantly enhanced wound healing in diabetic mice (Figure 3).

Mesenchymal cells isolated from adipose tissue represent a suitable target for cell-mediated gene therapy as they have been proven to be prone to viral-mediated gene transfer [41]. We then assessed whether overexpression of SDF-1, whose downmodulation plays a pivotal role in the pathophysiology of diabetic wounds, might result in a better therapeutic profile of the AT-SCs-mediated treatment. Indeed, we achieved further improvement by adenoviral-mediated SDF-1 gene transfer into AT-SCs before topical administration. Wound area and epithelial gap were significantly reduced in treated animals. In particular, the percentage of wound closure 3 days after injury was 24 in control mice 41 and 58%, respectively, in animals receiving AT-SCs and AT-SCs expressing SDF-1. At 5 days, values were 45, 68, and 78%, respectively. Full-thickness wounds were completely closed in 7 to 10 days in all AT-SCs-treated mice, while in controls, complete healing was achieved later than 14 days after the punch.

**Figure 4:**
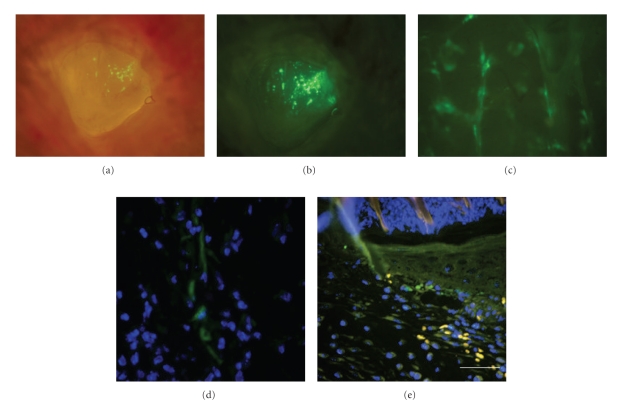
*Analysis of the wound area. *Gross examination under fluorescence microscope of wounds 5 days after administration of GFP-labelled cells. Original magnification 10X (a) and (b) and 40X (c). Anti GFP immunohistochemistry at the lesion site 2 (d) and 5 days post wounding (e) after administration of GFP labelled AT-SCs. Scale bar D–E 25 *μ*m.

By gross examination and immunohistochemical analysis, we determined the presence at the site of tissue regeneration of GFP-positive administered cells several days after administration (Figure 4). Further studies to determine biodistribution and persistence of administered cells were performed using *in vivo* bioluminescent imaging techniques (see below).

### 3.4. Tracking Transplanted AT-SCs by BLI

After *in vivo* administration of AT-SCs expressing luciferase, we monitored luciferase expression by real time *in vivo* imaging in the whole animal, together with a light photograph, to provide for anatomical references. The intensity of the signal detected by *in vivo* imaging can be precisely quantified and correlates with the presence of luciferase-expressing cells, and therefore with effective cell engraftment after administration. Since luciferin metabolism requires ATP, only living cells expressing luciferase are able to produce a signal. The ability to perform repeated analysis on the same animal at different time points allowed us to determine the kinetic of AT-SCs biodistribution and engraftment at the wound site.

**Figure 5:**
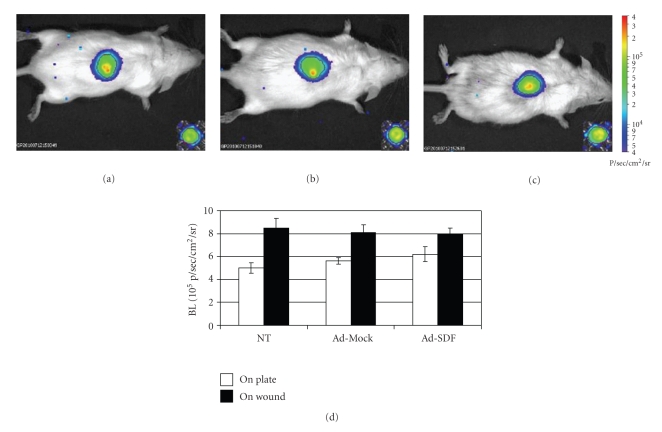
*Ex vivo and in vivo imaging of AT-SCs expressing luciferase at time 0. *Freshly isolated AT-SCs from luciferase-expressing mice were subjected to adenoviral-mediated gene transfer ((a) non-transduced; (b) Ad-mock-transduced; (c) SDF-1-transduced cells). The AT-SCs were placed in a 96-well cell culture plate and BLI imaging was performed (insert of each figure panel). The same cells were then administered to hyperglycemic mice right after performing a dorsal lesion and BLI was repeated. Panel (d) shows quantification of the signals. *Note.* The diameter of a well of a 96-well plate is 6.4 mm; the diameter of the biopsy punch used to induce wounding is 3.5 mm. Data are expressed as mean ± SEM indicated by error bars; *n* = 3 for each group.

AT-SCs were isolated from inguinal fad pads from luciferase-expressing mice.  Figure 5 shows the BLI signal originated from 7.5 × 10^5^ cells placed on a 96-well cell culture plate and of the same cells right after topical administration at the wound site. Quantification of the BLI signal did not show any statistically significant difference between non-transduced cells and AT-SCs transduced with adenoviral vectors expressing GFP and SDF-1 (Figure 5) at the time of administration. Differences in BLI signal observed from cells placed in a 96 well tissue culture and from the same cells after administration to the dorsum of the mouse are dependent, at least in part, on the approximately 3-fold difference in the area of a 96-well compared to the area of the biopsy punch.

**Figure 6:**
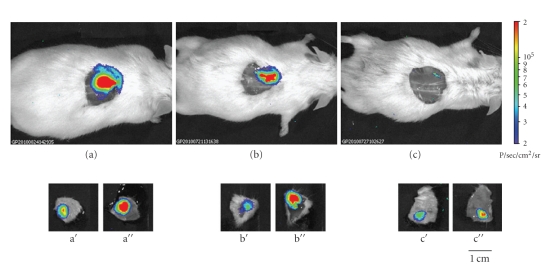
*Biodistribution after topical administration at the wound site of AT-SCs expressing luciferase. *BLI imaging of representative animals sacrificed 1 (a), 10 (b), and 15 (c) days after topical administration of luciferase-expressing cells at the wound site (*n* = 3). The region surrounding the wound was excided and BLI performed both with the skin facing the CCD camera (a′, b′, and c′) and on the opposite orientation (a′′, b′′, and c′′). At all time points, cells are mainly located in the lesion area with minimal spreading from the site of topical administration.

To determine biodistribution of delivered cells, some animals were sacrificed 1, 10, and 15 days after wound induction and topical administration at the lesion site of AT-SCs expressing luciferase. BLI was performed before and after sacrifice of the mouse and dissection of the wounded area. As shown in Figure 6, cells were mainly located at the lesion site, with minimal spreading from the site of delivery. Moreover, performing BLI on the dissected tissue with the skin facing the CCD camera produced a signal that was consistently lower than the one obtained with flipping the piece (Figure 6). This reflects the fact that administered cells mainly contributed to dermal and subdermal tissue regeneration and engrafted only in small part in the skin. We detected a positive signal in the region of the skin and in the corresponding subdermal area even at day 15, when the wound was entirely healed (Figure 6(c)). This clearly indicates engraftment and permanence of administered cells in the regenerated tissue even after complete healing.

**Figure 7:**
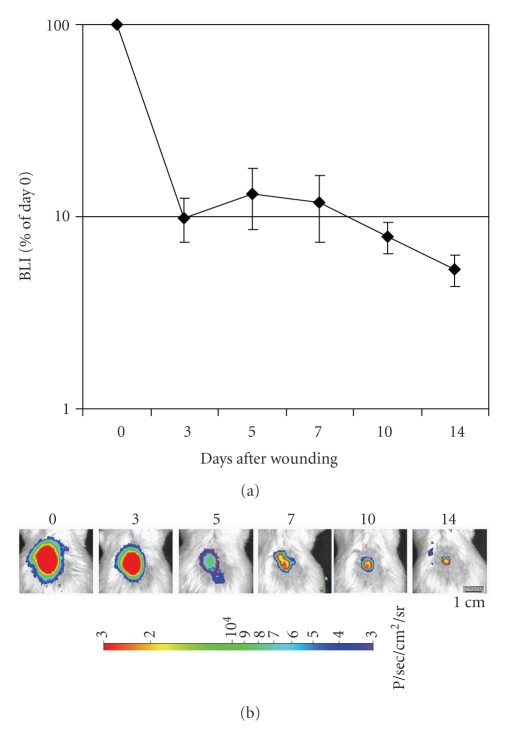
*Persistence and engraftment of AT-SCs expressing luciferase after topical administration at the wound. *Diabetic mice were treated after wounding with AT-SCs isolated from luciferase-expressing mice. Longitudinal analysis performed by BLI at different time points after the treatment indicates persistence of signal throughout the process of wound healing. Representative images from an experimental animal at different time points (*n* = 6). Data are indicated as the percentage of the value immediately after topical administration of the cells (day 0). Values are expressed as mean ± SEM indicated by error bars.

The kinetics of engraftment of administered cells, expressed as percent of the BLI signal at day 0 is reported on Figure 7, and it inversely correlates with the kinetics of wound healing as observed in Figure 3. Most of the signal associated with transplanted cells was lost between day 1 and day 3 after transplantation. A value of approximately 10% of the intensity observed at day 0 was maintained from day 3 to day 10, indicating cell engraftment. Although at a lower level, BLI signal was detectable also at 14 days after cell administration, suggesting cell engraftment at the site of tissue regeneration.

Dramatic reduction of stem cell survival after administration is the main obstacle hampering clinical translation of cell therapy protocols. In fact, up to 99% of grafted cells may die within the first few days of transplantation due to the rigors of the host microenvironment they are transferred in. This is mainly characterized by short supply of oxygen or nutritive substrates and by a massive excess of free radicals [42]. Several strategies to promote donor cell survival have been evaluated [43]. In particular, preconditioning of mesenchymal stem cells by treatment with the recombinant SDF-1 resulted in enhanced survival and proliferation under anoxic conditions *in vitro* and *in vivo* [44].

**Figure 8:**
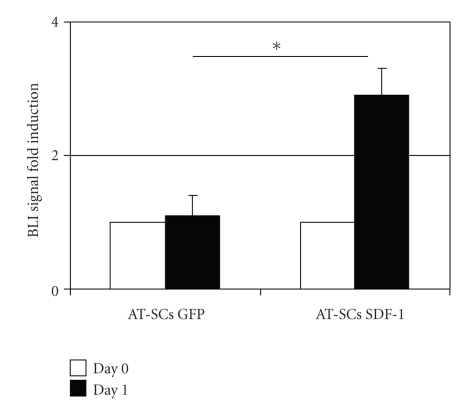
*Topically administration of AT-SCs expressing luciferase and overexpressing SDF-1. *SDF-1 overexpression promotes enhanced survival/proliferation of administered cells at the lesion site 1 day after wounding and cell administration, as assessed by BLI. Values are expressed as mean ± SEM indicated by error bars; *n* = 3, *P < .001.

The SDF-1*α*/CXCR4 ligand/receptor axis modulates several pivotal biological functions, including increased cell growth, proliferation, migration, survival, antiapoptosis, and transcriptional activation. In particular, it has been shown that overexpression of CXCR4 increases migration and proliferation of human adipose tissue-derived stromal cells [40]. As shown in Figure 3, we found that transplantation of AT-SCs overexpressing SDF-1 promotes wound healing in diabetic animals. Interestingly, we determined a statistically significant difference between BLI signals in animals receiving AT-SCs expressing SDF-1 relative to controls expressing GFP, 1 day after administration (Figure 8). This indicates that SDF-1 overexpression enhances survival/proliferation of administered cells at the lesion site at this time point, resulting in beneficial effects. Nonetheless, no statistically significant differences between the two groups were observed at later time points (3, 5, 7, and 14 days), suggesting that SDF-1 adenoviral-mediated overexpression failed to improve long-term cell engraftment in our experimental model. However, SDF-1 overexpression may promote prohealing effects to dermal fibroblasts and keratinocytes surrounding the lesion site [12, 21], resulting in improved healing observed in Figure 3. 

Our results show that AT-SCs administration at the lesion site improves wound healing in diabetic animals within days from wound induction. BLI imaging data indicate that a small fraction of administered cells engraft and participate in repairing the tissue at the lesion site. On the other hand, the amount of engrafted cells may be too low to fully explain the mechanisms of tissue regeneration. In addition, several evidences suggest that AT-SCs may exert their beneficial effects also via an autocrine effect promoting self-profileration and via a paracrine effects on dermal fibroblasts and keratonocytes surrounding the lesion site [21].

## 4. Conclusions

We have demonstrated that topical administration of AT-SCs improves impaired wound healing in diabetic mice. Moreover, using BLI, we were able to follow biodistribution and the kinetics of engraftment, survival, and proliferation of administered cells, which proved relatively permanent in the regenerated tissue even after complete healing. In addition, our data suggest that concomitant genetic manipulation of transplanted AT-SCs designed to promote overexpression of SDF-1 may further improve diabetic impaired wound healing.

## References

[1] O’Loughlin A, McIntosh C, Dinneen SF, O’Brien T (2010). Review paper: basic concepts to novel therapies: a review of the diabetic foot. *International Journal of Lower Extremity Wounds*.

[2] Jeffcoate WJ, Lipsky BA, Berendt AR (2008). Unresolved issues in the management of ulcers of the foot in diabetes. *Diabetic Medicine*.

[3] Barrientos S, Stojadinovic O, Golinko MS, Brem H, Tomic-Canic M (2008). Growth factors and cytokines in wound healing. *Wound Repair and Regeneration*.

[4] Wu Y, Zhao RCH, Tredget EE (2010). Concise review: bone marrow-derived stem/progenitor cells in cutaneous repair and regeneration. *Stem Cells*.

[5] Badillo AT, Redden RA, Zhang L, Doolin EJ, Liechty KW (2007). Treatment of diabetic wounds with fetal murine mesenchymal stromal cells enhances wound closure. *Cell and Tissue Research*.

[6] Fu X, Fang L, Li H, Li X, Cheng B, Sheng Z (2007). Adipose tissue extract enhances skin wound healing. *Wound Repair and Regeneration*.

[7] Badiavas EV, Falanga V (2003). Treatment of chronic wounds with bone marrow-derived cells. *Archives of Dermatology*.

[8] Zuk PA, Zhu M, Ashjian P (2002). Human adipose tissue is a source of multipotent stem cells. *Molecular Biology of the Cell*.

[9] Schäffler A, Büchler C (2007). Concise review: adipose tissue-derived stromal cells—basic and clinical implications for novel cell-based therapies. *Stem Cells*.

[10] Sterodimas A, de Faria J, Nicaretta B, Pitanguy I (2009). Tissue engineering with adipose-derived stem cells (ADSCs): current and future applications. *Journal of Plastic, Reconstructive and Aesthetic Surgery*.

[11] Rehman J, Traktuev D, Li J (2004). Secretion of angiogenic and antiapoptotic factors by human adipose stromal cells. *Circulation*.

[12] Kim WS, Park BS, Sung JH (2007). Wound healing effect of adipose-derived stem cells: a critical role of secretory factors on human dermal fibroblasts. *Journal of Dermatological Science*.

[13] Katz AJ, Tholpady A, Tholpady SS, Shang H, Ogle RC (2005). Cell surface and transcriptional characterization of human adipose-derived adherent stromal (hADAS) cells. *Stem Cells*.

[14] Planat-Benard V, Silvestre JS, Cousin B (2004). Plasticity of human adipose lineage cells toward endothelial cells: physiological and therapeutic perspectives. *Circulation*.

[15] Moon MH, Kim SY, Kim YJ (2006). Human adipose tissue-derived mesenchymal stem cells improve postnatal neovascularization in a mouse model of hindlimb ischemia. *Cellular Physiology and Biochemistry*.

[16] Sumi M, Sata M, Toya N, Yanaga K, Ohki T, Nagai R (2007). Transplantation of adipose stromal cells, but not mature adipocytes, augments ischemia-induced angiogenesis. *Life Sciences*.

[17] Miranville A, Heeschen C, Sengenès C, Curat CA, Busse R, Bouloumié A (2004). Improvement of postnatal neovascularization by human adipose tissue-derived stem cells. *Circulation*.

[18] Nakagami H, Maeda K, Morishita R (2005). Novel autologous cell therapy in ischemic limb disease through growth factor secretion by cultured adipose tissue-derived stromal cells. *Arteriosclerosis, Thrombosis, and Vascular Biology*.

[19] Lim JS, Yoo G (2010). Effects of adipose-derived stromal cells and of their extract on wound healing in a mouse model. *Journal of Korean Medical Science*.

[20] Ebrahimian TG, Pouzoulet F, Squiban C (2009). Cell therapy based on adipose tissue-derived stromal cells promotes physiological and pathological wound healing. *Arteriosclerosis, Thrombosis, and Vascular Biology*.

[21] Kim WS, Park BS, Sung JH (2009). The wound-healing and antioxidant effects of adipose-derived stem cells. *Expert Opinion on Biological Therapy*.

[22] Brem H, Tomic-Canic M (2007). Cellular and molecular basis of wound healing in diabetes. *Journal of Clinical Investigation*.

[23] Tepper OM, Carr J, Allen RJ (2010). Decreased circulating progenitor cell number and failed mechanisms of stromal cell-derived factor-1*α* mediated bone marrow mobilization impair diabetic tissue repair. *Diabetes*.

[24] Gallagher KA, Liu ZJ, Xiao M (2007). Diabetic impairments in NO-mediated endothelial progenitor cell mobilization and homing are reversed by hyperoxia and SDF-1*α*. *Journal of Clinical Investigation*.

[25] Badillo AT, Chung S, Zhang L, Zoltick P, Liechty KW (2007). Lentiviral gene transfer of SDF-1*α* to wounds improves diabetic wound healing. *Journal of Surgical Research*.

[26] Bermudez DM (2009). Inhibition of Stromal Derived Factor-1*α* (SDF-1*α*) in diabetic dermal wounds further prolongs the diabetic wound healing impairment. *Journal of Surgical Research*.

[27] Kortesidis A, Zannettino A, Isenmann S, Shi S, Lapidot T, Gronthos S (2005). Stromal-derived factor-1 promotes the growth, survival, and development of human bone marrow stromal stem cells. *Blood*.

[28] Zhang M, Mal N, Kiedrowski M (2007). SDF-1 expression by mesenchymal stem cells results in trophic support of cardiac myocytes after myocardial infarction. *FASEB Journal*.

[29] Zhao T, Zhang D, Millard RW, Ashraf M, Wang Y (2009). Stem cell homing and angiomyogenesis in transplanted hearts are enhanced by combined intramyocardial SDF-1*α* delivery and endogenous cytokine signaling. *American Journal of Physiology*.

[30] Okabe M, Ikawa M, Kominami K, Nakanishi T, Nishimune Y (1997). ’Green mice’ as a source of ubiquitous green cells. *FEBS Letters*.

[31] Cao YUA, Bachmann MH, Beilhack A (2005). Molecular imaging using labeled donor tissues reveals patterns of engraftment, rejection, and survival in transplantation. *Transplantation*.

[32] Di Rocco G, Iachininoto MG, Tritarelli A (2006). Myogenic potential of adipose-tissue-derived cells. *Journal of Cell Science*.

[33] Kern S, Eichler H, Stoeve J, Klüter H, Bieback K (2006). Comparative analysis of mesenchymal stem cells from bone marrow, umbilical cord blood, or adipose tissue. *Stem Cells*.

[34] Takashima T, Bonifacino JS, Dasso M, Harford JB, Lippincott-Schwartz J, Yamada KM (1999). Preparation and isolation of cells: preparing fibroblasts. *Current Protocols in Cell Biology*.

[35] He TC, Zhou S, Da Costa LT, Yu J, Kinzler KW, Vogelstein B (1998). A simplified system for generating recombinant adenoviruses. *Proceedings of the National Academy of Sciences of the United States of America*.

[36] Luo J, Deng ZL, Luo X (2007). A protocol for rapid generation of recombinant adenoviruses using the AdEasy system. *Nature Protocols*.

[37] Bilic G, Ochsenbein-Kölble N, Hall H, Huch R, Zimmermann R (2004). In vitro lesion repair by human amnion epithelial and mesenchymal cells. *American Journal of Obstetrics and Gynecology*.

[38] Passaniti A, Taylor RM, Pili R (1992). Methods in laboratory investigation: a simple, quantitative method for assessing angiogenesis and antiangiogenic agents using reconstituted basement membrane, heparin, and fibroblast growth factor. *Laboratory Investigation*.

[39] Cheng B, Liu HW, Fu XB, Sun TZ, Sheng ZY (2007). Recombinant human platelet-derived growth factor enhanced dermal wound healing by a pathway involving ERK and c-fos in diabetic rats. *Journal of Dermatological Science*.

[40] Cho HH, Kyoung KM, Seo MJ, Kim YJ, Bae YC, Jung JS (2006). Overexpression of CXCR4 increases migration and proliferation of human adipose tissue stromal cells. *Stem Cells and Development*.

[41] Morizono K, De Ugarte DA, Zhu M (2003). Multilineage cells from adipose tissue as gene delivery vehicles. *Human Gene Therapy*.

[42] Das R, Jahr H, van Osch GJ, Farrell E (2010). The role of hypoxia in bone marrow-derived mesenchymal stem cells: considerations for regenerative medicine approaches. *Tissue engineering. Part B, Reviews*.

[43] Haider HK, Ashraf M (2008). Strategies to promote donor cell survival: combining preconditioning approach with stem cell transplantation. *Journal of Molecular and Cellular Cardiology*.

[44] Pasha Z, Wang Y, Sheikh R, Zhang D, Zhao T, Ashraf M (2008). Preconditioning enhances cell survival and differentiation of stem cells during transplantation in infarcted myocardium. *Cardiovascular Research*.

